# Opioid treatment program-integrated facilitated telemedicine for hepatitis C treatment: a hybrid effectiveness-implementation analysis

**DOI:** 10.1186/s12906-025-05138-9

**Published:** 2025-10-15

**Authors:** Andrew H. Talal, Marianthi Markatou, Marija Zeremski, Anran Liu, Arpan Dharia, Saliyah G. George, Monika Taylor, Kathleen Davis, Avi Silber, Lawrence S. Brown, Jonathan N. Tobin

**Affiliations:** 1 Division of Gastroenterology, Hepatology, and Nutrition, Jacobs School of Medicine and Biomedical Sciences, University at Buffalo, State University of New York, 875 Ellicott Street, Suite 6090, Buffalo, NY 14203 USA; 2 Department of Biostatistics, University at Buffalo, Buffalo, NY USA; 3 Clinical Directors Network, Inc. (CDN), New York, NY USA; 4 Division of Liver Diseases, Department of Medicine, Icahn School of Medicine at Mount Sinai, New York, NY USA; 5 Crouse Hospital, Syracuse, NY USA; 6 Department of Psychiatry, University of Rochester, Rochester, NY USA; 7 Cornerstone Family Healthcare, Newburgh, NY USA; 8 START Treatment & Recovery Centers, Brooklyn, NY USA; 9 Center for Clinical and Translational Science, Rockefeller University, New York, NY USA

**Keywords:** Hepatitis C virus, HCV, Telemedicine, Opioid use disorder, Stepped wedge design, Pragmatic, Random forests, Coincidence analysis

## Abstract

**Background:**

Hybrid effectiveness-implementation designs evaluate the effectiveness and implementation of interventions. We retrospectively evaluated the implementation of a stepped-wedge cluster randomized controlled trial of a facilitated telemedicine model (experimental) integrated into opioid treatment programs (OTPs) compared to offsite referral (control) for hepatitis C virus (HCV) treatment. The trial period was March 2017–October 2022. We compared organizational and implementation characteristics associated with an HCV cure and with high satisfaction with healthcare delivery.

**Methods:**

We used the *R*each, *E*ffectiveness, *A*doption, *I*mplementation, *M*aintenance (RE-AIM) framework to guide data collection and evaluation. We evaluated the clinical effectiveness outcome (HCV cure) and patient-centered outcomes (changes between in-person and telemedicine patient satisfaction questionnaire subscales: Time Spent With Doctor and General Satisfaction). We evaluated 7 organizational and 16 implementation variables. We used random forests to obtain a list of variables with total importance weight of at least 95%. We subsequently conducted a configurational comparative method of coincidence analysis (CNA) to identify the variable combinations that are associated with the best outcomes.

**Results:**

The effectiveness of *reach* was enhanced by site identification of HCV RNA positive individuals. We found that low patient load per provider or counselor, site liaison presence, and high case manager availability increased clinical *effectiveness* (i.e., HCV cure). *Adoption and implementation*, assessed by high healthcare delivery satisfaction among participants in both arms, was associated with site liaisons, frequent case manager onsite presence and consistency, and low provider patient volume. Among telemedicine participants, onsite notifications and provider involvement in recruitment were additional variables associated with high healthcare satisfaction. In referral, providing patient education, low counselor patient volume, case manager involvement in site activities, and high case manager education levels were additional variables associated with high healthcare delivery satisfaction. Intervention *maintenance* has occurred at 10 sites.

**Conclusions:**

Compared to referral, facilitated telemedicine requires fewer variables for high effectiveness and patient satisfaction. The frequent onsite presence and consistency of the case manager and low provider and counselor volumes improved outcomes among both approaches. Improved outcomes among referral participants required more publicity, patient education, higher case manager education, more involvement in site activities, and occurred in university-affiliated sites.

**Trial registration:**

Clintrials.gov registration number NCT02933970; Comparison of Telemedicine to Usual Care for HCV Management for Methadone-maintained Individuals Full Text View ClinicalTrials.gov.

**Supplementary Information:**

The online version contains supplementary material available at 10.1186/s12906-025-05138-9.

## Background

Telemedicine has tremendous potential to increase healthcare access by overcoming temporal and geographic challenges to healthcare delivery. Implementation of telemedicine to underserved populations, however, necessitates consideration of issues, such as broadband connectivity, digital health literacy, and patient-provider trust [1, 2]. Addressing these issues, while simultaneously incorporating health equity principles, enables telemedicine to address health disparities [3]. Since data are limited on the approaches to implement telemedicine for underserved populations, especially in the context of a pragmatic clinical trial, we evaluated organizational and implementation factors that led to successful telemedicine implementation. We generated the data for this investigation from a pragmatic, randomized controlled trial (RCT) evaluating a facilitated telemedicine model for the treatment of hepatitis C virus (HCV) infection in opioid treatment programs (OTPs). Because of shared transmission routes, people with opioid use disorder (OUD) have the highest HCV incidence and prevalence [4]. Direct acting antiviral (DAA) medications for HCV have near 100% efficacy after a 2–3 month treatment course [5]. Based upon the therapeutic successes of DAAs, HCV elimination by 2030 has been promoted by several organizations [6–8]. Simultaneously, however, DAA uptake has been low as only one-third of treatment-eligible individuals in the United States received DAAs from 2013 to 2022 [9]. Without increasing access to curative HCV treatment, the objective of HCV elimination by 2030 will not be achieved.

The RCT was designed and powered as a pragmatic, comparative effectiveness trial of HCV treatment outcomes utilizing the stepped wedge design with multiple outcomes. We found that 90.3% of facilitated telemedicine participants (experimental condition) achieved a sustained virologic response (SVR-HCV cure) compared to 39.4% of those in offsite referral (usual care, control condition) odds ratio (OR) = 2.9 (p-value < 0.001; 95%confidence interval [CI] [2.0, 3.5] [10]. Additionally, participants were highly satisfied with facilitated telemedicine encounters, rated equivalent to in-person healthcare interactions [11]. The successful implementation of the pragmatic RCT necessitated development of strategies to conduct research in OTPs with no or limited research experience, as well as deploying approaches to engage an underserved population [12]. Furthermore, the RCT incorporated many attributes of well-designed pragmatic clinical trials, such as involving frontline staff in venues that are routine providers of OUD care [13]. We sought to evaluate the organizational and implementation characteristics required for successful implementation of a pragmatic clinical trial. Our specific area of investigation was implementation of facilitated telemedicine integrated into OTPs for HCV treatment. We evaluated two outcome measures: (1) HCV treatment effectiveness, defined by the percentage of individuals who achieved an SVR, and (2) patient satisfaction with healthcare delivery as assessed through components of the Patient Satisfaction Questionnaire (PSQ) [14].

## Methodological overview

###  Implementation science framework

We retrospectively employed random forests (RF) and coincidence analysis (CNA) to identify important characteristics and combinations of characteristics that led to successful facilitated telemedicine implementation and high satisfaction with healthcare delivery. The RCT period was March 2017–October 2022. We utilized the Reach, Effectiveness, Adoption, Implementation, and Maintenance (RE-AIM) framework [15, 16] to guide collection, analysis, and reporting of results of organizational and implementation characteristics that led to successful facilitated telemedicine implementation [17]. The primary clinical outcome was SVR defined as the absence of detectable HCV RNA in the serum 12 weeks post-treatment completion [5]. We also measured patient-centered outcomes with measures of general satisfaction (GS), time spent with doctor (TSWD), interpersonal manner (IM), and accessibility and convenience (AC).

We optimized the data collection according to the RE-AIM framework [18–21] (Supplemental Content 1.1, Table S1). We outlined patient, organizational, and implementation measures against the RE-AIM dimensions of reach, effectiveness, adoption, implementation, and maintenance. We examined available data sources and illustrated patient (Table 1), organizational (Table 1), and implementation characteristics (Table 2). Once these data were acquired and merged with individual patient-level measures, we conducted RF analysis [22] followed by CNA.

**Table 1:** Organizational characteristics

Organization Characteristic	Value	Range
Location		
New York Upstate, N (%)	6 (50%)	
New York Downstate, N (%)	6 (50%)	
Opioid treatment program affiliation		
University/Hospital, N (%)	4 (33.3%)	
Free Standing, N (%)	4 (33.3%)	
Health System, N (%)	4 (33.3%)	
Opioid treatment program size		
Small (< 400 patients), N (%)	1 (8.3%)	
Medium (400–700 patients), N (%)	8 (66.7%)	
Large (> 700 patients), N (%)	3 (25%)	
Percentage of rural patients		
Urban, N (%)	6 (50%)	
Urban/Suburban/Rural, N (%)	6 (50%)	
Rural, mean %	18%	0% − 38%
Opioid treatment program staffing		
N patients per MD provider, mean	475	146–780
N patients per NP/PA provider, mean	436	219–671
N patient per counselor, mean	57	20–90

*Abbreviations*: *MD* Medical doctor, *NP* Nurse practitioner, *PA* Physician’s assistant

**Table 2:** Implementation characteristics

Implementation Characteristic	Value
Health information technology characteristics	
On-site services and staffing	
Phlebotomy available on-site	
Yes, for all patients, N (%)	7 (58.3%)
Yes, from third study period (telemedicine only), N (%)	1 (8.3%)
Only for study patients, N (%)	2 (16.7%)
No, N (%)	2 (16.7%)
Electronic reminders for direct acting antiviral dispensing at methadone dispensing window, N (%)	7 (58.3%)
Site liaison provided to facilitate study	8 (66.7%)
Case manager fulltime equivalent	
Days spent on-site	
4–5, N (%)	7 (58.3%)
1–4, N (%)	5 (41.7%)
Turnover - number of case managers per site	
1, N (%)	6 (50%)
2, N (%)	1 (8.3%)
3, N (%)	5 (41.7%)
Education	
Bachelor’s or associate degree	5 (41.7%)
Master’s or other post-graduate degree	7 (58.3%)
Patient/stakeholder engagement	
Sites with 100% attendance at PAC meetings	9 (75%)
Sites with 93% attendance at PAC meetings	3 (25%)
Implementation Strategies	
Patients educated about the study, yes %	100%
Verbally by Case Manager	75%
Using liver model	33%
Poster at OTP with contact info	33%
Open forum discussion	33%
Staff engagement by case manager, yes %	100%
Attend staff meetings	67%
Informational presentation	33%
MD and/or PA support	50%
Targeted conversations	33%
Case Manager attended patients group sessions, yes %	75%
“Flag” alerts at methadone dispensing window, yes %	83%

*Abbreviations:* *PAC* Patient advisory committee, *MD* Medical doctor, *PA* Physician’s assistant, *OTP* Opioid treatment program

###  RE-AIM framework definitions and applications

#### Reach

Reach is defined as “the intervention reaching the target population” [23]. We used the consent rate, defined as the percentage of approached, eligible participants who consented to RCT participation, as an estimate of reach at the patient level. In our case, the RCT was conducted entirely in OTPs that dispense methadone (Supplemental Content 1.1.1).

#### Effectiveness

We measured clinical effectiveness as the number of individuals with undetectable HCV RNA 12 weeks post-treatment completion, i.e., those who achieved an SVR and are considered cured. Supplemental Content 1.1.2 contains information on SVR rates and rates per site are illustrated (Table S2) [10].

#### Adoption

A conventional definition of adoption is “to what extent are those to whom the intervention is targeted participating”. Since effectiveness is a direct measure of adoption, we rephrased the definition as “what are the factors that affect participation in the intervention by the target population?”

In prior investigation, we found that empathy was the most important evaluation factor when people with OUD rate their satisfaction with healthcare delivery [11] (Table S3). In the RCT, we assessed healthcare delivery satisfaction twice, at the first and final encounters. For this analysis, we evaluated the ratio of responses from the second (i.e., the SVR timepoint) measurement over the initial (i.e., the initial participant visit) among the 344 participants who completed the PSQ at both timepoints. We illustrate the means of the ratios computed per site according to PSQ subscales stratified by study arm (Table S4). From the PSQ data, we evaluated the three subscales: TSWD, IM, and AC. Since TSWD had the highest weight of the three, we prioritized TSWD outcomes to measure adoption. Similarly, since IM had the second-highest weight of the three and AC is an often-cited benefit of collocated care [24], we include the analytical results of IM and AC in the supplemental content. Supplemental Content 1.1.3 presents the approach to calculating PSQ ratios. A list of PSQ outcome variables is illustrated (Table S5).

#### Implementation

Implementation has been defined as “to what extent was the intervention continuously implemented?” We evaluated organizational and implementation characteristics associated with GS.

#### Maintenance

At the organizational level, we defined maintenance as “to what extent did onsite HCV care, either through facilitated telemedicine or some component of the intervention, become routine within the OTPs”, consistent with the definition used by prior investigators [23]. At the individual level, we measured maintenance as the durability of SVR as indicated by the number of reinfections that occurred during the two-year follow-up period [10].

###  Quantitative analysis

#### Data acquisition and preparation

We collected data from all 12 RCT-participating sites, and we coded the available measures (Tables 1 and 2, S1) into dichotomous categories. After exclusion of specific variables (Supplemental Content 1.2 and 1.3), the data included 23 features and 10 outcome variables. The features were divided into 7 organizational (Table S6) and 16 implementation (Table S7) variables.

#### Random forests (RF) analysis

RF identifies variables that are most important for a given outcome. The RF-assigned scores indicate their importance in predicting a given outcome. We used the model-independent permutation feature importance evaluation method to obtain the importance score of each variable. Specifically, we used Python 3.9.12, the machine learning library “scikit-learn”, function “RandomForestRegressor” for fitting RF models [25, 26]. We next ranked the variables from highest to lowest importance using the function “permutation_importance” until the variable list accounted for total importance weight of at least 95%. These selected variables form a subset of all the variables. We used 500 trees, and each feature was permuted 100 times. Definitions of frequent terms are listed in Supplemental Content 1.6.

#### Coincidence Analysis (CNA)

CNA is a causal inference method that provides “solution pathways” or “strategy configurations” that are associated with the best outcomes. The procedure identifies combinations of conditions that are minimally sufficient for an outcome or multiple causal pathways to achieve the best outcomes [22]. We assessed the following through CNA: [1] consistency (i.e., proportion of cases with an outcome condition covered by a solution compared to all cases covered by a configuration) [2], coverage (i.e., proportion of cases with the outcome covered by the solution compared to all cases with the outcome), and [3] complexity (i.e., the number of conditions in a specific configuration). We examined these parameters (a) separately for the facilitated telemedicine intervention versus offsite referral to determine which factors are associated with improved outcomes and (b) those that differed between the two study arms.

To select the configurations, we ran the CNA using the “minimally sufficient condition” function within the R package “cna”, version 3.5.1 [27, 28]. This function provides a condition table that lists all the configurations that meet the consistency threshold, with columns listing the outcome, condition, consistency, and coverage. We start with consistency 1 and coverage 0.25 and obtain the configurations. We then repeat this process by reducing the consistency by 0.05 on each iteration. Eventually, we collect all the configurations that meet consistency of greater or equal to 0.75 and coverage of greater or equal to 0.25. These conditions range in complexity from 1 to 4. Beginning with complexity 1, we next select the first reasonable condition with highest coverage in each complexity. The total number of solutions per each outcome of interest is shown in Supplemental Content 1.4, Tables S8-S11.

## Results

### Organizational characteristics

All participating OTPs were in New York State, six downstate and six upstate. Organizational characteristics of 12 participating OTPs are illustrated (Tables 1 and S1). Four OTPs were university-affiliated, four were health system-affiliated, and four were community-based. Upstate sites had between 9% and 38% of participants who resided in rural areas, younger aged patients, and fewer minority patients compared with downstate sites.

### RE-AIM evaluation

We used the RE-AIM framework (Table S1) to guide the identification and evaluation of the factors important for implementation of facilitated telemedicine into OTPs (Table 3). In our description below, we initially describe the organizational variables and subsequently the implementation variables.

**Table 3:**
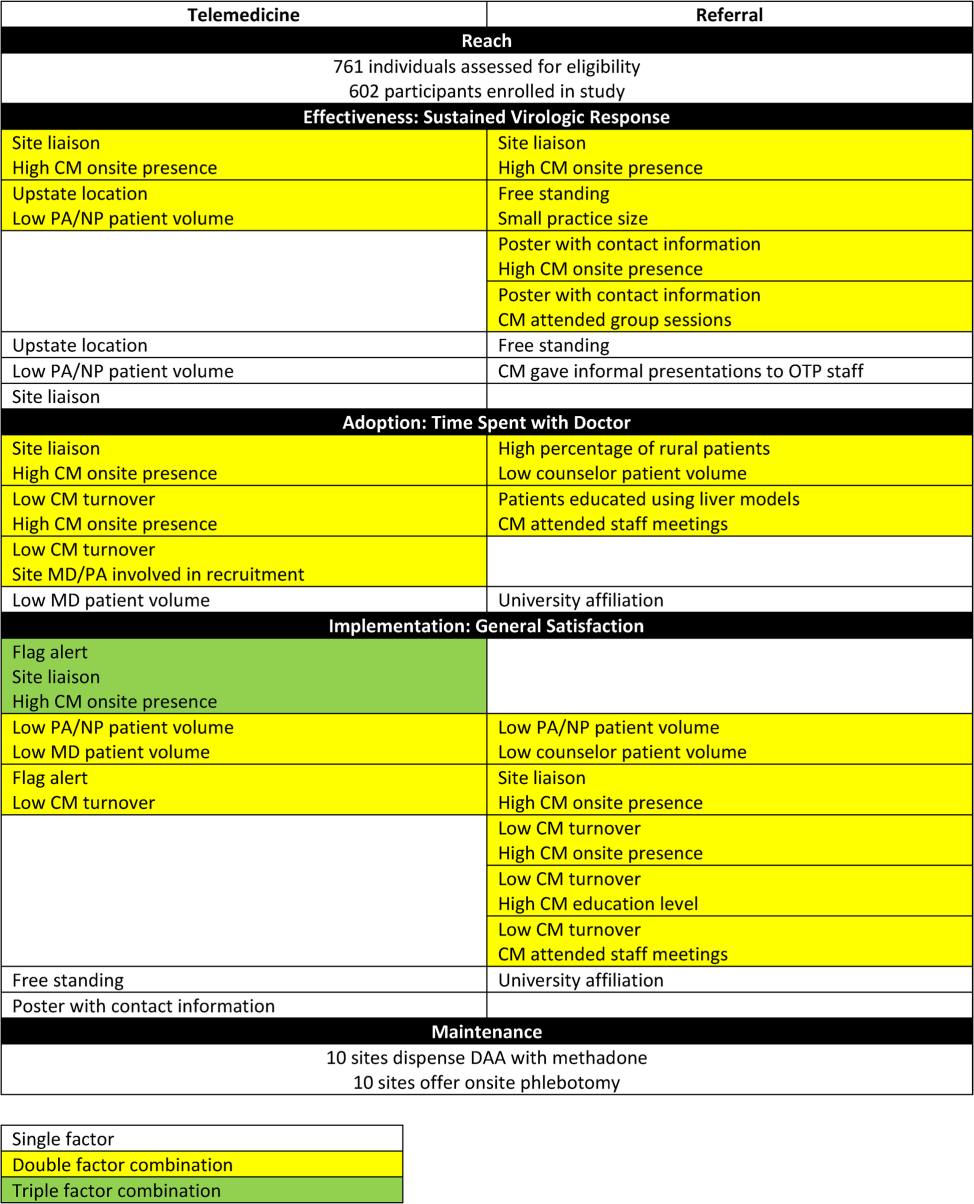
Evaluation of organizational and implementation characteristics by RE-AIM (Reach, Effectiveness, Adoption, Implementation, Maintenance) components

Table 3 depicts organizational and implementation characteristics as they relate to the Reach, Effectiveness, Adoption, Implementation, and Maintenance (RE-AIM) framework. The quantitative evaluation is supported by Random Forests-Coincidence Analysis for Effectiveness, Adoption, and Implementation.

*Abbreviations: CM* Case manager, *NP* Nurse practitioner, *PA* Physician assistant, *OTP *Opioid treatment program, *MD* Medical doctor, *DAA* Direct-acting antiviral

### Reach

In the RCT, 761 participants were assessed for eligibility and 602 were enrolled, 96.5% of the original sample size of 624 participants. A total of 159 participants were ineligible for the study. The major reason for study ineligibility among 144 individuals was no HCV treatment indication. We estimated that approximately 1414 HCV antibody positive individuals were approached at upstate sites, and we enrolled 311 (22.0%).

Recruitment approaches differed across the 12 sites. As the downstate sites assessed HCV RNA annually, most patients were eligible for HCV treatment, and CMs readily approached HCV RNA positive individuals. To engage potential upstate participants, CMs had to publicize the RCT using a variety of techniques, making an accurate assessment of the number of people approached infeasible.

### Effectiveness

Overall, the intention-to-treat SVR rate in the telemedicine arm was 90.3% and 39.4% in the referral arm with an estimated logarithmic odds ratio of 2.9 (p-value < 0.001; 95%CI [2.0, 3.5]). Among telemedicine arm participants, we identified ‘upstate location’, ‘low nurse practitioner (NP)/physician assistant (PA) patient volume’, and ‘site liaison’ as important single factors (Table 3). We also obtained two important combinations: (1) ‘Upstate location’ + ‘Low PA/NP patient volume’ and (2) ‘Site liaison’ + ‘High CM onsite presence’. ‘Upstate location’ and ‘low NP/PA patient volume’ are organization characteristics, while ‘site liaison’ and ‘high CM onsite presence’ are implementation characteristics.

Among referral participants, we identified two single factors, ‘free standing’ and ‘CM gave informal presentations’. We identified four combination factors: (1) ‘free-standing’ + ’small practice size’ (2), ‘site liaison’ + ‘high CM onsite presence’ (3), ‘high CM onsite presence’ + ‘poster with contact information’, and (4) ‘CM attended group sessions’ + ‘poster with contact information’ (Table 3). ‘Free standing’ and ‘small practice size’ are organization characteristics, while ‘CM gave informal presentations’, ‘site liaison’, ‘high CM onsite presence’, ‘poster with contact information’ and ‘CM attended group sessions’ are implementation characteristics.

OTP staff in practices with fewer patients had the potential advantage of delivering greater patient-centered care. In terms of staffing ratios, upstate practices typically had lower patient volumes. Some upstate OTPs had multiple providers with provider-to-patient ratios ranging from 146 to 780 patients for medical doctors (MDs), 219 to 671 for NPs or PAs, and 20–75 patients per counselor. At the downstate sites, each MD/PA/NP covered 410 to 539 patients and 46 to 90 per counselor.

### Adoption

In prior work, we found that Time Spent with Doctor (TSWD) was the most highly weighted PSQ subscale [11]. Therefore, TSWD is an important adoption measure to identify the factors associated with higher levels of satisfaction with healthcare delivery. In the telemedicine arm, when assessing TSWD, three important combinations identified were: (1) ‘Site liaison’ + ‘High CM onsite presence’ (2), ‘Low CM turnover’ + ‘High CM onsite presence’, and (3) ‘Low CM turnover’ + ‘Site MD/PA involved in recruitment’. The only important single factor was ‘low MD patient volume’ (Table 3). ‘Low MD patient volume’ is an organization characteristic, while ‘site liaison’, ‘high CM onsite presence’, ‘low CM turnover’ and ‘site MD/PA involved in recruitment’ are implementation characteristics. For important factor combinations for Interpersonal Manner (IM) and Accessibility and Convenience (AC) in telemedicine participants, please see Supplemental Content 1.5 (Table S12).

In the referral arm, when assessing TSWD, two important combinations identified were: (1) ‘High percentage of rural patients’ + ‘Low counselor patient volume’ and (2) ‘Patients educated using liver models’ + ‘CM attended staff meetings’. The only important single factor was ‘university affiliation’ (Table 3). ‘High percentage of rural patients’, ‘low counselor patient volume’ and ‘university affiliation’ are organization characteristics, while ‘patients educated using liver models’ and ‘CM attended staff meetings’ are implementation characteristics. For important factor combinations for IM and AC in referral participants, please see Supplemental Content 1.5 (Table S12).

### Implementation

We used the PSQ subscale of GS to assess implementation. In the telemedicine arm, we identified three important combinations: (1) ‘Low PA/NP patient volume’ + ‘Low MD patient volume’ (2), ‘Flag alert’ + ‘Low CM turnover’, and (3) ‘Flag alert’ + ‘Site liaison’ + ‘High CM onsite presence’. The important single factors were ‘free standing’ and ‘poster with contact information’ (Table 3). ‘Low MD/PA/NP patient volume’ and ‘free standing’ are organization characteristics, while ‘flag alert’, ‘low CM turnover’, ‘site liaison’, ‘high CM onsite presence’ and ‘poster with contact information’ are implementation characteristics.

In the referral arm, we identified five combinations: (1) ‘Low PA/NP patient volume’ + ‘Low counselor patient volume’ (2), ‘Site liaison’ + ‘High CM onsite presence’ (3), ‘High CM onsite presence’ + ‘Low CM turnover’ (4), ‘Low CM turnover’ + ‘High CM education level’, and (5) ‘Low CM turnover’ + ‘CM attended staff meetings’. The only important single factor was ‘university affiliation’ (Table 3). ‘Low PA/NP/counselor patient volume’ and ‘university affiliation’ are organization characteristics, while ‘site liaison’, ‘high CM onsite presence’, ‘low CM turnover’, ‘high CM education level’ and ‘CM attended staff meetings’ are implementation characteristics.

### Maintenance

The RCT offered hands-on experience with telemedicine to the participating OTPs. At some sites, facilitated telemedicine served as a conduit to direct onsite HCV management by providing onsite clinicians with requisite expertise to manage HCV. Since RCT completion, 10 of the 12 study sites (83%) report that they have continued to offer HCV treatment, either through telemedicine or onsite clinicians directly prescribing DAAs. These sites have continued to dispense HCV medications onsite together with methadone. In recent years, New York State has lifted HCV treatment restrictions to permit PAs, NPs, and non-specialists to prescribe DAAs. Ten of the sites have continued with onsite phlebotomy, a component of the facilitated telemedicine model.

## Discussion

Hybrid effectiveness-implementation studies can elucidate factors that lead to successful interventions. We conducted an RCT to evaluate the effectiveness of facilitated telemedicine compared to offsite referral for HCV treatment. In this investigation, we examined organizational and implementation characteristics that led to successful treatment outcomes and high patient satisfaction with healthcare delivery. We observed that factor combinations in facilitated telemedicine were parsimonious, suggesting that facilitated telemedicine requires fewer factors for successful implementation, which can accelerate HCV treatment uptake. Indeed, the duration between the initial visit and HCV treatment initiation was significantly shorter in facilitated telemedicine (mean [SD] 49.9 [48.1] days) compared to referral (mean [SD] 123.5 [92.4] days) [10]. In referral, ‘university affiliation’, ‘high CM onsite presence’, and ‘low CM turnover’ were particularly frequent, important variables. Obtaining buy-in and support from OTP leadership and staff were vital for RCT conduct. Important to both treatment approaches was the presence of a site liaison, a dedicated staff person acting as a point-of-contact for implementation of RCT procedures. Sites that lacked a site liaison at times suffered from disjointed teams. However, given RCT integration into OTP workflows and transferability of duties, even sites without site liaisons performed well.

Two common themes underlying many of the other important variable combinations we identified are trust and OTP staff workload. For example, ‘CM attended group sessions’ indicates that CMs sought to engage with OTP patients in a safe space with the goal of health improvement [29]. ‘Low MD, PA/NP and counselor patient volume’ and ‘High CM onsite presence’ both indicate the availability of the site staff and CMs, respectively, to address enrollment, retention, and RCT procedures. Similarly, variables such as ‘Flag alert’, ‘CM attended staff meetings’, and ‘Site MD/PA involved in recruitment’ are factors that foster integration of CMs into OTP workflows and promote cross-discipline collaboration. Taken together, these factors underscore the importance of clear communication and frequent interactions between OTP patients and staff with CMs to achieve both successful therapeutic effectiveness and high satisfaction with healthcare delivery. CM onsite presence and involvement in OTP activities were critical to engaging and supporting patients in HCV care. This notion is supported by the important findings of high CM onsite presence, low CM turnover, and CM attending staff meetings. Furthermore, CM integration into OTP workflows fostered frequent communication with staff and patients that assisted CMs in identifying and mitigating study-related issues and supporting patients in real-time.

The implementation of facilitated telemedicine aligned with the missions of the OTP as well as that of the New York State Office of Addiction Services and Supports, the state agency that oversees OTPs. A recent guidance has promoted patient-centered healthcare within OTPs [30]. Many of the important characteristics identified here promote patient-centered care, including augmenting OTP staff and patient HCV knowledge. Increasing knowledge about addiction, HCV, and health systems was foundational to achieving high patient satisfaction. Improved knowledge enabled staff to advocate for patients throughout the HCV treatment course. One approach to increase staff education is Project Echo [31]. Understanding opioid addiction was a prerequisite to building trust and respect with people with OUD. From the patients’ perspective, improved HCV-related knowledge, specifically concerning the treatment course, assuaged anxiety. Patient-centered care, specifically time to address patient concerns and clear communication channels, also increased patient satisfaction. Similarly, the importance of ‘High CM education level’ suggests the ability of CMs to satisfactorily explain the offsite referral process. In the referral arm, the university affiliation of the OTP supports a straightforward referral process, which could also include sharing an electronic health record, thereby improving patient accessibility and convenience and provider communication. Other investigators have shown that people with OUD frequently evade negative perceptions of OUD shame, which appears to be a major reason why referral arm participants avoided or delayed HCV care [32, 33].

Many important implementation and organizational characteristics involve CM availability and participation in OTP activities. We found three important factors, ‘Low CM turnover’, ‘Poster with contact information’ and ‘High CM onsite presence’, that are associated with the accessibility and availability of CMs. Additionally, factors that reflected CM involvement in OTP activities, such as ‘CM attended group sessions’ and ‘CM attended staff meetings’ illustrate potential mechanisms through which CM involvement was important for high intervention effectiveness and satisfaction with healthcare delivery. Contemporaneously, OTP staff involvement was also important in supporting people with OUD through HCV treatment. Factors such as ‘Low MD, PA/NP, counselor patient volume’, ‘Site MD/PA involved in recruitment’ and ‘Flag alert’ (i.e., as a communication method) illustrate the symbiotic effects that CMs and OTP staff had on patient engagement and retention in care. Similarly, onsite phlebotomy removed patients’ transportation barriers to accessing care and provided check-ins and reminders. From qualitative interviews, we learned that warm handoffs and open communication channels are important strategies to enhance full integration of facilitated telemedicine into OTP workflows [34, 35].

Pragmatic trials are usually patient-centered, involve multiple stakeholders during all study phases, are usually conducted in venues that are routine healthcare providers for the target population, and emphasize effectiveness in real-world settings [13, 36]. In this study, we identified a variety of organizational and implementation features that support successful execution of a pragmatic trial among underserved populations. Some features, such as warm handoffs between counselors and case managers, and clinical care collaboration, are indicative of approaches utilized to fully integrate facilitated telemedicine into OTP workflows. Organizational factors, such as low provider-patient or counselor-patient volume, reflect the capacity of the OTP staff to dedicate time to addressing complementary healthcare conditions, such as HCV. Pragmatic research also enables assessment of multiple outcomes simultaneously, as we did in this investigation in which we evaluated HCV cure and components of satisfaction with healthcare delivery.

Strengths of this work include the use of emerging analytic methods (CNA after application of random forests) to assess variation in clinical and patient-centered outcomes as a function of patient, organizational, and implementation variables. We selected candidate variables identified through the RE-AIM framework using the random forests method, in which the variables with minimal importance to the outcomes were eliminated. Unlike traditional quantitative methods, CNA alone does not provide strong statistical inference and relies on subjective judgments by researchers for variable selection, threshold setting, and configurations. It is also sensitive to small changes in the data, which could substantially alter the results by adding or removing variables from the combinations. The use of random forests is a strength because it avoids subjective variable selection for use in CNA. Furthermore, the use of two different types of outcome variables, SVR as a clinical effectiveness outcome and PSQ subscales (TSWD and GS) as patient-centered measures of satisfaction with healthcare delivery, enabled us to focus on innovative aspects of facilitated telemedicine as a healthcare delivery modality. A limitation is the retrospective evaluation of organizational and implementation characteristics. Some RE-AIM measures were acquired late in the RCT, after its completion, or not at all. We were unable to obtain independent data on in-person communication between potentially eligible patients and study staff, and the fidelity of telemedicine-based HCV treatment in real-world settings other than OTPs. For example, in the calculation of Reach, we had to estimate the number of people approached for study participation at the upstate sites. Downstate sites maintained lists of HCV RNA positive patients.

## Conclusions

The findings presented in this manuscript have important implications for future research. We have elucidated that selected approaches apply to facilitated telemedicine, offsite referral, or both. We found that fewer variables are required for successful implementation of facilitated telemedicine than referral. In referral, university-affiliated OTPs and high case manager education levels were important attributes. Common variables to both approaches were attributes that promote trust and consider OTP workloads. We found that opportunities to establish trusting relationships with patients were important to achieving successful clinical and patient-centered outcomes. Patient-centeredness has been gaining increasing recognition [30], and is promoted by cross-disciplinary collaboration, clear communication, and frequent interactions. Pragmatic trials offer opportunities to evaluate implementation characteristics in real-world clinical settings and to study the influences of approaches on OTP workflows. Understanding factors of effective facilitated telemedicine targeted to underserved populations can support future research among people with socioeconomical vulnerabilities and low digital health literacy. It can also expand participation in telemedicine as a healthcare delivery approach.

## Supplementary Information

Supplementary Material 1.

Supplementary Material 2.

## Data Availability

The datasets generated and analyzed during the current study are not publicly available due to confidentiality concerns but are available from the corresponding author on reasonable request.
