# Supplementary material for: Opioid treatment program-integrated facilitated telemedicine for hepatitis C treatment: a hybrid effectiveness-implementation analysis
Source: BMC Complement Med Ther. 2025 Oct 15;25:377. doi: 10.1186/s12906-025-05138-9 (PMC12522442; doi:10.1186/s12906-025-05138-9)
Supplement: Supplementary file 1 — Supplementary Material 1. [file 12906_2025_5138_MOESM1_ESM.docx]

**Supplemental Online Content**

- 1. **Outcome variables**

Categorization of variables according to the RE-AIM (Reach, Effectiveness, Adoption, Implementation, Maintenance) framework at the patient and organizational levels [1, 2].

|  | **Measure Definition** | |
| --- | --- | --- |
|  | **Implementation Variables** | **Organizational Variables** |
| **Reach** | In-person communication between potentially eligible patients and study staff | Study participation rate among HCV positive patients |
|  | Consent rate | Staff engagement: case managers attended staff meetings |
|  | | |
| **Effectiveness** | Undetectable HCV RNA test at 3 months post-treatment | Rate of study participants who achieved SVR (3 months post-treatment) |
|  | Patient satisfaction | % of patients who used >90% of HCV medications |
|  | | |
| **Adoption** | % of patients who had initial telemedicine consultation with liver specialist | % of NYS OTPs who will adopt telemedicine for HCV management |
|  | % of patients who initiated HCV treatment (telemedicine) | Site liaison provided to facilitate study |
|  |  | MD and/or PA support (MD/PA involved in the recruitment process) |
|  | | |
| **Implementation** | % of patients who completed HCV treatment (100% medications) | Fidelity of telemedicine-based HCV treatment in real-world settings |
|  | % of expected telemedicine visits patient completed with liver specialist | Remote access to spoke EHR |
|  |  | Electronic DOT reminders |
|  |  | Phlebotomy on-site |
|  |  | Adaptation: |
|  |  | CM turnover |
|  |  | Number of days/weeks CM spent on-site |
|  |  | CM attended patient group sessions |
|  |  | CM provided informational presentations to site staff |
|  |  | CM conducted targeted conversations with site staff |
|  |  | Organization continues to offer HCV treatment (via telehealth or on-site) after trial |
|  | | |
| **Maintenance** | Reinfection rate | Organization continues to offer HCV treatment, either through telemedicine or onsite as well as model components after trial completion. |

Table S1: Table S1 illustrates the entire scope of variables sought for inclusion in the analysis categorized by availability (green) and unavailability (red). Variables were categorized according to patient or organizational level and to the RE-AIM framework.

Abbreviations: HCV, hepatitis C virus; RNA, ribonucleic acid; SVR, sustained virologic response; NYS, New York State; OTP, opioid treatment program; MD, medical doctor; PA, physician assistant; EHR, electronic health record; DOT, directly observed therapy; CM, case manager.

- - 1. **Calculation of number of potentially eligible individuals**

The number of potentially eligible individuals from downstate sites was readily retrievable since they maintained lists of HCV RNA-positive individuals and conducted HCV RNA testing annually. For the upstate sites, we estimated the numbers of HCV-infected individuals at each site who might have been HCV-treatment eligible at the initiation of the RCT. For this estimation, we initially multiplied the practice size by the percentage of HCV antibody positive individuals (provided by either the New York State agency that coordinates a network of opioid treatment programs [i.e., Office of Addiction Services and Supports {OASAS} or the participating sites themselves). From this number, we sought to estimate the number of HCV antibody positive individuals without detectable HCV RNA, which can occur either through prior HCV treatment or spontaneous resolution. With regard to prior treatment, recent data have shown that SVR occurred in only 34% of HCV-infected individuals in the United States during the first decade of DAAs [3]. Additionally, in chronic HCV infection, spontaneous resolution occurs at 0.36% per person-years of follow up, a very rare event [4]. Therefore, we took a conservative approach and subtracted 20% from the estimated percentage of HCV antibody positive individuals in each upstate program to account for prior treatment and/or spontaneous HCV resolution during the chronic phase.

**1.1.2 Sustained virologic response** **rates**

The sustained virologic response (SVR) rates are obtained based on the 602 patients who enrolled in the study. The following table lists the number of patients and their SVR status within each site for both arms.

For each arm, the SVR rate of a site is computed as the number of patients who have SVR status “yes” divided by the total number of patients in the corresponding arm of that site. For example, the SVR rate of site 1 in the referral (Ref) arm is computed as 7/(7 + 0 + 6) = 53.85%. The SVR rate in Ref and SVR rate in telemedicine are the two SVR outcomes.

| Site | Referral SVR | Telemedicine SVR |
| --- | --- | --- |
| 1 | 53.8% | 97.4% |
| 2 | 33.3% | 92.3% |
| 3 | 35.9% | 100% |
| 4 | 30.8% | 92.1% |
| 5 | 53.8% | 92.3% |
| 6 | 41.0% | 92.3% |
| 7 | 23.1% | 92.3% |
| 8 | 30.7% | 78.6% |
| 9 | 23.1% | 61.5% |
| 10 | 34.6% | 70.6% |
| 11 | 19.2% | 92.3% |
| 12 | 61.5% | 97.4% |
| **Total** | **35.3%** | **90.7%** |

Table S2: Sustained virologic response outcomes per site.

Abbreviations: SVR, sustained virologic response.

**1.1.3 Ratios of Patient Satisfaction Questionnaire (PSQ)**

In the PSQ outcomes, we are looking at four subcategories: 1) Time Spent with Doctor (TSWD); 2) Interpersonal Manner (IM); 3) General Satisfaction (GS); 4) Accessibility & Convenience (AC).

The patients we are focusing on are the 344 patients who completed the questionnaires at both time points 1 (T1) and 2 (T2).

The questions within each subcategory are listed as follows:

TSWD: Time Spent with Doctor

Q12: Those who provide my Hep C care sometimes hurry too much when they treat me.

Q15: My Hep C doctor usually spends plenty of time with me.

IM: Interpersonal Manner

Q10: My Hep C doctor acts too businesslike and impersonal toward me.

Q11: My Hep C doctor treats me in a very friendly and courteous manner.

AC: Accessibility & Convenience

Q8: I have easy access to the Hep C doctor I need.

Q9: Where I get my Hep C care, people have to wait too long for their treatment.

Q16: I find it hard to get an appointment for Hep C care right away.

Q18: I am able to get Hep C care whenever I need it.

GS: General Satisfaction

Q3: The Hep C care I have been receiving is just about perfect.

Q17: I am dissatisfied with some things about the Hep C care I receive.

The responses for each question were processed so that scores range from 1 to 5 with 5 corresponding to the highest satisfaction and 1 to the lowest satisfaction.

The answer to the questions included in the aforementioned subcategories were obtained at two timepoints. We used the ratio of the score a particular question received at the second time point over the score of the same question obtained at the first time point as our outcome.

For each subcategory, we assign different weights to the ratios described above. We used the method of Lagrange multiplier[5] to find the optimal weights for each question so that the weighted variance in each subcategory is minimized. The obtained weights for the different ratios of the question scores under each subcategory are as follows:

|  | Ratio of Question Scores | Weight |
| --- | --- | --- |
| TSWD | R12 | 0.318 |
|  | R15 | 0.682 |
| IM | R10 | 0.045 |
|  | R11 | 0.955 |
| GS | R3 | 0.739 |
|  | R17 | 0.261 |
| AC | R8 | 0.681 |
|  | R9 | 0.007 |
|  | R16 | 0.109 |
|  | R18 | 0.203 |

Table S3: Weights for each question per Patient Satisfaction Questionnaire Subcategory of interest

Abbreviations: TSWD, time spent with doctor; IM, interpersonal manner; GS, general satisfaction; AC, accessibility and convenience.

For each patient, we first compute his/her score at T2 over T1, then we obtain the weighted ratio for each subcategory. Next compute the means of the ratios of all the patients within each site.

For example, if a patient has Q12 = 3 & Q15 = 5 at T1, and Q12 = 4 & Q15 = 5 at T2, his ratio for Q12 is 4/3 = 1.333 and ratio for Q15 is 5/5 = 1. Then his weighted TSWD score will be 0.318 × 1.333 + 0.682 × 1 = 1.106.

The following table lists the means of the ratio computed on each site, and the 8 columns correspond to the 8 PSQ subscales we included in the analysis:

Table S4: Means of the ratios computed per site according to Patient Satisfaction Questionnaire subscales of interest stratified by study arm.

Abbreviations: UC, usual care or referral; Tel, telemedicine; TSWD, time spent with doctor; IM, interpersonal manner; GS, general satisfaction; AC, accessibility and convenience.

All the 10 outcome variables were processed into binary variables (high/low) using their medians for the configurational comparative methods (CCM) analyses:

| Variable name | Variable type | Possible outcomes |
| --- | --- | --- |
| Sustained virologic response rate | Binary | Sustained virologic response-cure   - Referral high if >= 32.69% - Referral low if < 32.69% - Telemedicine high if >= 84.62% - Telemedicine low if < 84.62% |
| Time spent with doctor | Binary | - Referral high if >= 1.0629 - Referral low if < 1.0629 - Telemedicine high if >= 1.0535 - Telemedicine low if < 1.0535 |
| Interpersonal manner | Binary | - Referral high if >= 0.9978 - Referral low if < 0.9978 - Telemedicine high if >= 1.0227 - Telemedicine low if < 1.0227 |
| General satisfaction | Binary | - Referral high if >= 1.0729 - Referral low if < 1.0729 - Telemedicine high if >= 1.0370 - Telemedicine low if < 1.0370 |
| Accessibility and convenience | Binary | - Referral high if >= 1.0317 - Referral low if < 1.0317 - Telemedicine high if >= 1.0449 - Telemedicine low if < 1.0449 |

Table S5: List of patient satisfaction questionnaire outcome variables

- 1. **Organizational variables**

The final list of organizational variables used in the analysis is illustrated (Table S6).

| Variable name | Variable type | Possible outcomes |
| --- | --- | --- |
| Location | Binary | - 1 if upstate - 0 if downstate |
| OTP affiliation | Categorical | - 1 if university - 2 if free standing - 3 if health system / hospital |
| Practice size | Binary | - 1 if number of patients >= 514 - 0 if number of patients < 514 |
| Percentage of rural patients | Categorical | - 1 if percentage is 0% - 2 if percentage <= 13% - 3 if percentage > 13% |
| MD patient volume | Binary | - 1 if number of patients >= 488 - 0 if number of patients < 488 |
| NP/PA patient volume | Binary | - 1 if number of patients >= 412 - 0 if number of patients < 412 |
| Counselor patient volume | Binary | - 1 if number of patients >= 53 - 0 if number of patients < 53 |

Table S6: Organizational characteristics.

Abbreviation: OTP, opioid treatment program; NP, nurse practitioner; PA, physician assistant; MD, medical doctor.

- 1. **Implementation variables**

The final list of implementation variables used in the analysis is illustrated (Table S7). Variables that were excluded from the final analysis because they were present in all sites (i.e., they did not show variability) included:

1. Broadband strength,
2. Secure space for medications onsite,
3. Attendance at patient advisory committee meetings,
4. Substance Abuse Mental Health Services Administration (SAMHSA) brochure
5. Flexible scheduling for study appointments
6. Appointment reminder calls to patients
7. Dedicated office hours in the opioid treatment program

Variables that we judged not to impact the outcomes based upon stakeholders' input included:

1. Electronic health record type-only impacted the provider, not the patient.
2. Remote access to electronic health record-only impacted the provider, not the patient.
3. Telehealth platform-judged to have insufficient differences between platforms.
4. Specialty pharmacy available on site-judged not to affect patient medication delivery.
5. Dried blood spot tests-only implemented during the follow up period to assess for reinfections as it is approved as a “research use only” test.
6. Electronic reminders for direct acting antiviral dispensing at methadone dispensing window-are seen only by the nurse and are not evaluated by the patients themselves.
7. Patient demographics and insurance-judged not to affect patient medication delivery.

| **Variable name** | **Variable type** | **Possible outcomes** |
| --- | --- | --- |
| Phlebotomy on-site | Binary | - 1 if yes - 0 if no |
| Electronic reminders for directly observed therapy available at methadone dispensing window | Binary | - 1 if yes - 0 if no |
| Flag alert “on hold” | Binary | - 1 if yes - 0 if no |
| Site liaison | Binary | - 1 if yes - 0 if no |
| Number of days per week case manager present on-site (recruitment period) | Binary | - 1 if averaged number of days >= 4 - 0 if averaged number of days < 4 |
| Case manager turnover | Binary | - 1 if number of case manager >= 3 - 0 if number of case manager < 3 |
| Case manager education | Binary | - 1 if case manager’s highest degree is master’s degree or equivalent - 0 if case manager’s highest degree is bachelor’s degree or equivalent |
| Case manager attended patient group sessions | Binary | - 1 if yes - 0 if no |
| Patients verbally educated by case manager | Binary | - 1 if yes - 0 if no |
| Patients educated using liver model (by case manager) | Binary | - 1 if yes - 0 if no |
| Poster at opioid treatment program with contact info | Binary | - 1 if yes - 0 if no |
| Case manager had open forum discussions with patients | Binary | - 1 if yes - 0 if no |
| Case manager attended site staff meetings | Binary | - 1 if yes - 0 if no |
| Case manager gave informal presentations to OTP staff | Binary | - 1 if yes - 0 if no |
| Case manager had targeted conversations with site staff | Binary | - 1 if yes - 0 if no |
| Site physician and/or physician assistant were involved in recruitment process | Binary | - 1 if yes - 0 if no |

Table S7: Implementation factors.

**1.4 Total number of solutions for each outcome of interest**

| **Arm** | **Complexity** | **Number of solutions via 2-step** | **Total solutions via 2-step** |
| --- | --- | --- | --- |
| **Referral** | 1 | 0 | 14 |
|  | 2 | 11 |  |
|  | 3 | 3 |  |
|  | 4 | 0 |  |
| **Telemedicine** | 1 | 10 | 36 |
|  | 2 | 26 |  |
|  | 3 | 0 |  |

Table S8: Total number of solutions provided by the 2-step method for sustained virological response aggregated over all pairs (consistency, coverage). Two-step is a method consists of random forest analysis followed by coincidence analysis. The results are presented per complexity level.

Abbreviations: CNA, coincidence analysis; TWSD, time spent with doctor; IM, interpersonal manner; GS, general satisfaction; AC, accessibility & convenience.

| **Arm** | **Complexity** | **Number of solutions via 2-step** | **Consistency** | **Coverage** |
| --- | --- | --- | --- | --- |
| **Referral** | 1 | 1 | 0.75 | 0.500 |
|  | 2 | 10 | 1 | 0.333 |
|  | 3 | 0 | 1 | 0.500 |
|  | 4 | 0 | 1 | 0.333 |
| **Telemedicine** | 1 | 3 | 1 | 0.750 |
|  | 2 | 0 | 1 | 0.750 |
|  | 3 | 0 | 1 | 0.375 |

Table S9: Number of solutions provided by 2-step method for sustained virological response. The solutions have the highest consistency and coverage per level of complexity and across methods. The two-step method consists of random forest analysis followed by coincidence analysis.

Abbreviations: CNA, coincidence analysis; TWSD, time spent with doctor; IM, interpersonal manner; GS, general satisfaction; AC, accessibility & convenience.

| **Arm** | **Outcome** | **Complexity** | **Number of solutions via 2-step** | **Total solutions via 2-step** |
| --- | --- | --- | --- | --- |
| **Referral** | TSWD | 1 | 2 | 46 |
|  |  | 2 | 34 |  |
|  |  | 3 | 10 |  |
|  |  | 4 | 0 |  |
|  | IM | 1 | 1 | 53 |
|  |  | 2 | 42 |  |
|  |  | 3 | 10 |  |
|  |  | 4 | 0 |  |
|  | GS | 1 | 3 | 36 |
|  |  | 2 | 28 |  |
|  |  | 3 | 5 |  |
|  | AC | 1 | 2 | 32 |
|  |  | 2 | 30 |  |
|  |  | 3 | 0 |  |
| **Telemedicine** | TSWD | 1 | 2 | 36 |
|  |  | 2 | 29 |  |
|  |  | 3 | 5 |  |
|  |  | 4 | 0 |  |
|  | IM | 1 | 2 | 36 |
|  |  | 2 | 26 |  |
|  |  | 3 | 8 |  |
|  |  | 4 | 0 |  |
|  | GS | 1 | 2 | 36 |
|  |  | 2 | 24 |  |
|  |  | 3 | 9 |  |
|  |  | 4 | 1 |  |
|  | AC | 1 | 4 | 54 |
|  |  | 2 | 32 |  |
|  |  | 3 | 18 |  |

Table S10: Total number of solutions provided by 2-step method for satisfaction with healthcare delivery and aggregated over all pairs of (consistency, coverage). The two-step method consists of random forest analysis followed by coincidence analysis. The results are presented per complexity level.

Abbreviations: CNA, coincidence analysis; TWSD, time spent with doctor; IM, interpersonal manner; GS, general satisfaction; AC, accessibility & convenience.

| **Arm** | **Outcome** | **Complexity** | **Number of solutions via 2-step** | **Consistency** | **Coverage** |
| --- | --- | --- | --- | --- | --- |
| **Referral** | TWSD | 1 | 1 | 1 | 0.500 |
|  |  | 2 | 6 | 1 | 0.500 |
|  |  | 3 | 0 | 1 | 0.500 |
|  |  | 4 | 0 | 1 | 0.333 |
|  | IM | 1 | 1 | 0.750 | 0.500 |
|  |  | 2 | 4 | 1 | 0.666 |
|  |  | 3 | 0 | 1 | 0.833 |
|  |  | 4 | 0 | 1 | 0.666 |
|  | GS | 1 | 1 | 1 | 0.333 |
|  |  | 2 | 5 | 1 | 0.500 |
|  |  | 3 | 0 | 1 | 0.500 |
|  | AC | 1 | 0 | 1 | 0.500 |
|  |  | 2 | 1 | 1 | 0.666 |
|  |  | 3 | 0 | 1 | 0.500 |
| **Telemedicine** | TSWD | 1 | 0 | 1 | 0.500 |
|  |  | 2 | 7 | 1 | 0.500 |
|  |  | 3 | 1 | 1 | 0.500 |
|  |  | 4 | 0 | 1 | 0.333 |
|  | IM | 1 | 2 | 0.75 | 0.500 |
|  |  | 2 | 1 | 1 | 0.666 |
|  |  | 3 | 8 | 1 | 0.333 |
|  |  | 4 | 0 | 1 | 0.333 |
|  | GS | 1 | 1 | 1 | 0.500 |
|  |  | 2 | 6 | 1 | 0.500 |
|  |  | 3 | 1 | 1 | 0.500 |
|  |  | 4 | 1 | 1 | 0.333 |
|  | AC | 1 | 0 | 1 | 0.500 |
|  |  | 2 | 3 | 1 | 0.666 |
|  |  | 3 | 0 | 1 | 0.666 |

Table S11: Number of solutions provided by 2-step method. The solutions have the highest consistency and coverage per level of complexity and across methods. The 2-step is a method consisting of random forest analysis followed by coincidence analysis.

Abbreviations: CNA, coincidence analysis; TWSD, time spent with doctor; IM, interpersonal manner; GS, general satisfaction; AC, accessibility & convenience.

**1.5 Results**

Interpersonal manner: In the telemedicine arm, we identified ‘University/Health system affiliation’ as being important. We also identified the combination of ‘high CM onsite presence’ + “low CM turnover’. When assessing IM in referral, we identified the combinations of (1) ‘small practice size’ + ‘low MD patient volume’ and (2) ‘onsite phlebotomy’ + ‘high CM onsite presence’. (Table S12).

Accessibility and convenience: In the telemedicine arm, the important single factors were ‘low MD patient volume’ and ‘site MD/PA involved in recruitment’. We also identified ‘high onsite CM presence’ + ‘site liaison’ as an important combination. In the referral arm, we identified the combinations of (1) ‘low PA/NP patient volume’ + ‘low counselor patient volume’ and (2) ‘low CM turnover’ + ‘CM attended site staff meetings’ as important.

| **Telemedicine** | | **Referral** | |
| --- | --- | --- | --- |
| **Interpersonal Manner** | | | |
| High CM onsite presence | Low CM turnover | High CM onsite presence | Phlebotomy onsite |
| University/Health system affiliation | | Small practice size | Low MD patient volume |
| **Accessibility and Convenience** | | | |
| Site liaison | High CM onsite presence | Low PA/NP patient volume | Low counselor patient volume |
| Site MD/PA involved in recruitment | | Low CM turnover | CM attended staff meetings |
| Low MD patient volume | |  | |

| Single factor |
| --- |
| Double factor combination |

Table S12 depicts organizational and implementation characteristics for Interpersonal Manner and Accessibility and Convenience as identified through the Random Forest-Coincidence Analysis.

Abbreviations: CM, case manager; MD, medical doctor; NP, nurse practitioner; PA, physician assistant.

**1.6 Definitions**

Sustained virologic response: The absence of detectable HCV RNA in the blood three months post treatment completion is designated as a cure or sustained virological response (SVR).

Site clinician: Each site had a medical doctor (MD), nurse practitioner (NP), or physician assistant (PA) who was primarily responsible for treatment of opioid use disorder. As part of the randomized controlled trial, these individuals were present during telemedicine encounters to assist with the physical examination.

Site liaison: Some sites had a dedicated staff person acting as a point-of-contact for implementation of study procedures.

Flagged alerts: Prompts from the electronic medical record to alert a provider that a medical intervention was needed when participants presented for methadone dispensing.

**References**

1. Damschroder LJ, Aron DC, Keith RE, Kirsh SR, Alexander JA, Lowery JC. Fostering implementation of health services research findings into practice: a consolidated framework for advancing implementation science. Implement Sci. 2009;4:50.

2. Damschroder LJ, Hagedorn HJ. A guiding framework and approach for implementation research in substance use disorders treatment. Psychol Addict Behav. 2011;25(2):194-205.

3. Wester C, Osinubi A, Kaufman HW, Symum H, Meyer WA, 3rd, Huang X, et al. Hepatitis C Virus Clearance Cascade - United States, 2013-2022. MMWR Morb Mortal Wkly Rep. 2023;72(26):716-720.

4. Bulteel N, Partha Sarathy P, Forrest E, Stanley AJ, Innes H, Mills PR, et al. Factors associated with spontaneous clearance of chronic hepatitis C virus infection. J Hepatol. 2016;65(2):266-272.

5. Beavis B, Dobbs IM: "Static Optimization". Optimization and Stability Theory for Economic Analysis. New York: Cambridge University Press; 1990.
